# Intraoperative microelectrode recording under general anesthesia guided subthalamic nucleus deep brain stimulation for Parkinson's disease: One institution's experience

**DOI:** 10.3389/fneur.2023.1117681

**Published:** 2023-02-23

**Authors:** Kang Qian, Jiajing Wang, Jing Rao, Peng Zhang, Yaqiang Sun, Wenqing Hu, Jie Hao, Xiaobing Jiang, Peng Fu

**Affiliations:** ^1^Department of Neurosurgery, Union Hospital, Tongji Medical College, Huazhong University of Science and Technology, Wuhan, China; ^2^Wuhan National Laboratory for Optoelectronics, Britton Chance Center for Biomedical Photonics, Huazhong University of Science and Technology, Wuhan, China; ^3^Institute of Automation, Chinese Academy of Sciences, Beijing, China; ^4^Guangdong Institute of Artificial Intelligence and Advanced Computing, Guangzhou, China

**Keywords:** general anesthesia, local anesthesia, microelectrode recording, subthalamic nucleus deep brain stimulation, Parkinson's disease

## Abstract

**Objective:**

Microelectrode recording (MER) guided subthalamic nucleus deep brain stimulation (STN-DBS) under local anesthesia (LA) is widely applied in the management of advanced Parkinson's disease (PD). Whereas, awake DBS under LA is painful and burdensome for PD patients. We analyzed the influence of general anesthesia (GA) on intraoperative MER, to assess the feasibility and effectiveness of GA in MER guided STN-DBS.

**Methods:**

Retrospective analysis was performed on the PD patients, who underwent bilateral MER guided STN-DBS in Wuhan Union Hospital from July 2019 to December 2021. The patients were assigned to LA or GA group according to the anesthetic methods implemented. Multidimensional parameters, including MER signals, electrode implantation accuracy, clinical outcome and adverse events, were analyzed.

**Results:**

A total of 40 PD patients were enrolled in this study, including 18 in LA group and 22 in GA group. There were no statistically significant differences in patient demographics and baseline characteristics between two groups. Although, the parameters of MER signal, including frequency, inter-spike interval (ISI) and amplitude, were obviously interfered under GA, the waveforms of MER signals were recognizable and shared similar characteristics with LA group. Both LA and GA could achieve effective electrode implantation accuracy and clinical outcome. They also shared similar adverse events postoperatively.

**Conclusion:**

GA is viable and comparable to LA in MER guided STN-DBS for PD, regarding electrode implantation accuracy, clinical outcome and adverse events. Notably, GA is more friendly and acceptable to the patients who are incapable of enduring intraoperative MER under LA.

## Introduction

Parkinson's disease (PD) is one of the most common neurodegenerative diseases, characterized by resting tremor, rigidity, bradykinesia, postural instability and gait disturbance (1). Bilateral subthalamic nucleus deep brain stimulation (STN-DBS) that relieves motor complications as well as non-motor symptoms, has become a cornerstone in the management of advanced PD during the past decades (2, 3).

Generally, the procedure of electrode implantation is implemented under local anesthesia (LA), allowing for intraoperative microelectrode recording (MER) and test stimulation during DBS surgery (1, 3). Indeed, there are several advantages, when STN-DBS is performed under LA. Since STN could not be visualized directly in the past, intraoperative MER and test stimulation were employed to identify the effective therapeutic target, to compensate for the brain shift and to reduce the stimulation-related side effects (1, 4). Up to now, LA is still the preferred anesthetic method to perform STN-DBS in most centers. Intraoperative MER and test stimulation have become the standard procedures in DBS surgery (4). However, awake DBS under LA is painful and burdensome for PD patients. They have to withstand the clinical testing and surgical procedures with a prolonged period of off-medication, suffering from anxiety and exhaustion. Moreover, the patients are required to wear a stereotactic frame on their head and endure the entire surgical procedures with the frame fixed to the operation table, which may result in intolerable pain and psychological sequelae (5, 6). There are also increased risks of hemorrhage and infection, if unintended large motions occur during the surgery (1, 7). Notably, patients with extreme anxiety, reduced cooperation, severe convulsions and difficult breathing are incapable of enduring awake DBS under LA (1, 8).

Owning to these concerns of awake DBS under LA, it has been a growing trend to perform STN-DBS under general anesthesia (GA) (7, 9, 10). Advances in magnetic resonance imaging (MRI) techniques have made it possible to visualize STN directly and enable MRI-guided STN-DBS to emerge as an alternative to conventional surgery (11). Likewise, intraoperative MER can also be performed to verify the neurophysiological target under GA (12). Implementation of MER guided STN-DBS surgery under GA can shorten operative time and improve patient comfort (4). However, it remains controversial whether GA interferes with MER signals, electrode implantation accuracy, clinical outcome and adverse events. To shed further light on these key issues, we performed a retrospective cohort study to assess the influence of anesthetic methods on MER guided STN-DBS.

## Methods

### Patients and clinical assessment

**Figure 1:**
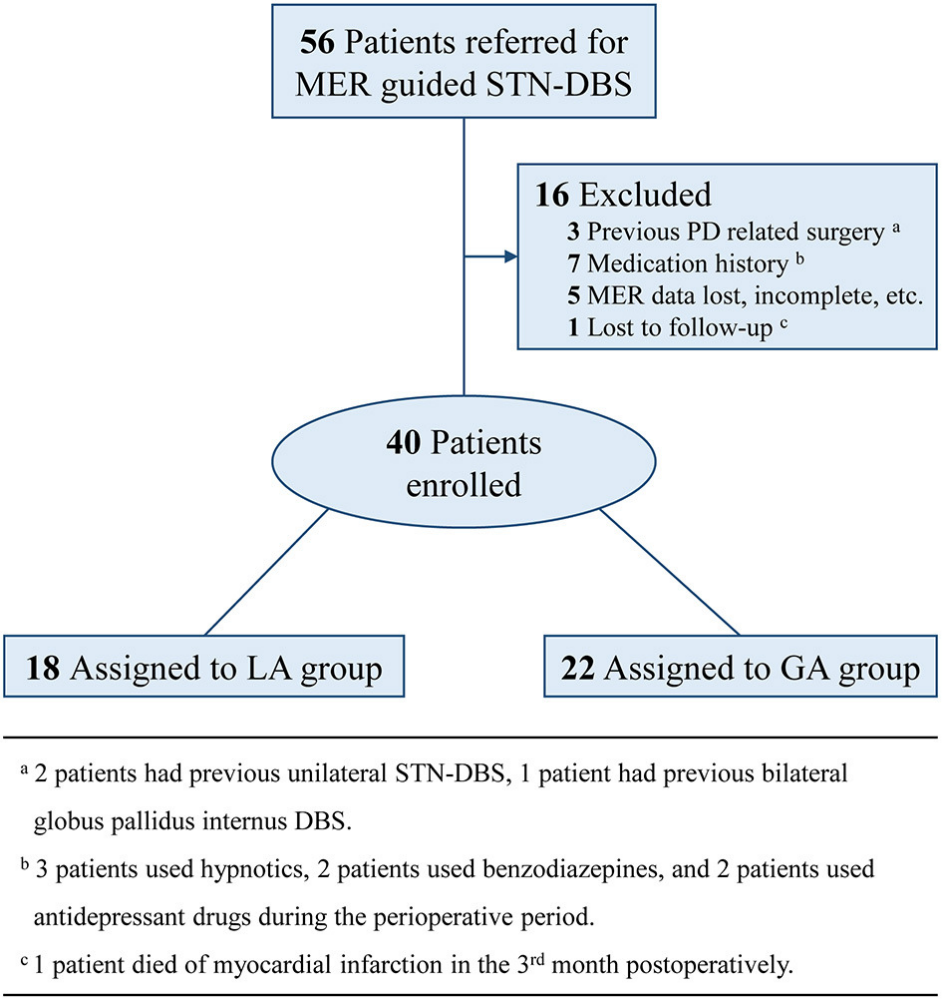
Consort flow diagram.

PD patients who were referred to our center for bilateral MER guided STN-DBS from July 2019 to December 2021, were included in this study. Exclusion criteria were: (a) previous PD related surgery; (b) medication history, which might have influences on MER signals; (c) MER data lost, incomplete, or with quality problems; (d) lost to follow-up within 6 months. The consort flow diagram was presented as Figure 1. All patients included in this study were preoperatively assessed with the application of Unified Parkinson Disease Rating Scale III (UPDRS-III), Hoehn and Yahr (H&Y) Staging, and Mini Mental State Examination (MMSE) under the condition of off-medication, which was defined as medication discontinued over 12 h. Preoperative levodopa equivalent daily dose (LEDD) was also recorded as the baseline.

### Anesthetic management

The anesthetic method was determined mainly according to the patients' preferences. Whereas, patients who were incapable of enduring awake DBS, including those with extreme anxiety, reduced cooperation, severe convulsions and difficult breathing, were operated under GA. We also showed reasons for the patients who were operated under GA in Supplementary Table 1. According to the anesthetic method applied, patients were assigned to LA group and GA group. In LA group, patients received local scalp anesthesia with 0.5% ropivacaine and kept conscious without sedation during MER and electrode implantation. In GA group, patients were administered a bolus of 2 mg/kg BW propofol and 1 mg/kg BW remifentanil for induction. Then, anesthesia was maintained at 2 mg/kg BW propofol and 1 mg/kg BW remifentanil by a target-controlled infusion (TCI) system. Bispectral index (BIS) was applied to monitor the depth of anesthesia. The infusion of anesthetics was adjusted before MER and BIS was maintained at 40–60 to ensure the recognition of STN signals.

### Surgical procedures

All patients are required to take a preoperative 3-T MRI scan and a stereotactic frame-based computed tomography (CT) scan. Then, the CT image was co-registered with MRI image to determine the target site, the trajectory angles (arc angle, ring angle) and the target coordinates (X_1_, Y_1_, and Z_1_). The X_1_, Y_1_ and Z_1_ coordinates were defined as the distance from the target site to the midpoint of anterior commissure-posterior commissure (AC-PC) line in medial-lateral (X-), anterior-posterior (Y-) and superior-inferior (Z-) axes respectively. The planned trajectories were kept away from intracranial vessels according to MRI images.

When the surgical preparation was finished, bilateral burr holes were made. MERs were performed in 1 to 3 trajectories per side and started at 6 mm above the planned target depth. The trajectory with the maximum length of STN was selected as the optimal trajectory and the site with the strongest STN signal was selected as the implantation site. Only in LA group, intraoperative test stimulations were implemented to reduce the stimulation-related symptoms and side effects. Subsequently, the quadripolar DBS electrode was implanted into the confirmed target and connected with the implantable pulse generator (IPG). Postoperative CT scan was applied to verify the electrode placement and exclude hemorrhage in 24 h.

### MER analysis

MER signals in the target where DBS electrode was implanted in were selected for analysis. MER signals were band-pass filtered (Butterworth filter) at 300–6000 Hz and sampled at a rate of 24 kHz. Only MER signals that had stable activities significantly over the baseline amplitude of background noise were selected and a threshold was applied to detect the spikes. The spike sorting for a single unit was performed by NeuroExplorer (version 5.311). The MER parameters, including spike frequency, inter-spike interval (ISI), minimum and maximum value of waveform, were recorded for further analysis. The borders of STN were identified according to the changes in firing patterns and the length of STN was recorded for further analysis.

### Outcome analysis

The outcome analysis included electrode implantation accuracy evaluation and postoperative clinical assessment. Postoperative CT image was co-registered with preoperative MRI image to evaluate the electrode implantation accuracy. The coordinates of electrode implantation site (X_2_, Y_2_, and Z_2_) were defined as the distance from the center of implanted electrode to the midpoint of AC-PC line in X-, Y-, and Z-axes. Vector errors (ΔX=|X_1_-X_2_|, ΔY=|Y_1_-Y_2_|, ΔZ=|Z_1_-Z_2_|, mm) and Euclidean distance ([ΔX^2^ + ΔY^2^ + ΔZ^2^]^1/2^, mm) were employed to assess the distance between the electrode implantation site to the intended target site, yielding the electrode implantation accuracy. The length of STN was also applied to verify the suitable target. Postoperative clinical assessment was performed in 6 months. UPDRS (-I, -II, -III, -IV) was applied to assess the motor and non-motor symptoms under on-DBS/off-medication condition. Postoperative LEDD was recorded and compared with the baseline. Reduction in LEDD was regarded as a parameter demonstrating the efficacy of STN-DBS. Within 24 h postoperatively, the patients' experiences were assessed using Visual Analog Scale (VAS) and Kolcaba General Comfort Questionnaire (GCQ). The adverse events were also recorded for further analysis.

### Statistical analysis

Statistical analysis was conducted with the use of SPSS software, version 26.0 (IBM Corp). Continuous variables with normal distribution were evaluated by Student's *t*-test and described as mean value with standard deviation (SD). Categorical variables were evaluated by chi-square test and presented as frequency with percentage (%). *P*-value < 0.05 was considered as statistically significant difference.

## Results

### Demographics and baseline characteristics

A total of 40 PD patients who experienced bilateral MER guided STN-DBS in our center, were enrolled in this study. Among these cases, 18 patients who experienced STN-DBS under LA, were assigned to LA group and 22 patients who experienced STN-DBS under GA, were assigned to GA group. The LA and GA groups have similar demographics and baseline characteristics of patients, including sex, age at onset of PD, age at surgery, duration of PD, UPDRS-III (off-medication), H&Y stage (off-medication), MMSE (off-medication) and LEDD (*P* > 0.05). These results were presented in Table 1.

**Table 1:** Demographics and baseline characteristics of PD patients.

	**LA (*n* = 18)**	**GA (*n =* 22)**	***P*** **-value**
Male, No. (%)	8 (44.4)	6 (27.3)	0.26
Female, No. (%)	10 (55.6)	16 (72.7)	0.26
Age at onset of PD (year), mean±SD	55.8 ± 4.0	52.8 ± 7.5	0.14
Age at surgery (year), mean±SD	63.2 ± 6.0	60.8 ± 7.4	0.27
Duration of PD (year), mean±SD	8.0 ±2.4	8.0 ± 2.8	0.96
UPDRS-III (med off), mean±SD	50.2 ± 12.4	51.5 ± 11.3	0.74
**H&Y stage (med off), No. (%)**			
1	0 (0)	0 (0)	-
2	6 (33.3)	11 (50.0)	0.29
3	10 (55.6)	9 (40.9)	0.36
4	2 (11.1)	2 (9.1)	0.83
5	0 (0)	0 (0)	-
MMSE (med off), mean±SD	28.6 ± 0.9	28.05 ± 1.5	0.21
LEDD (mg/day), mean±SD	1491.7 ± 393.4	1527.3 ± 413.1	0.78

UPDRS-III, unified Parkinson's disease rating scale III; H&Y stage, Hoehn and Yahr stage; MMSE, mini mental state examination; LEDD, levodopa equivalent daily dose.

### MER analysis

**Figure 2:**
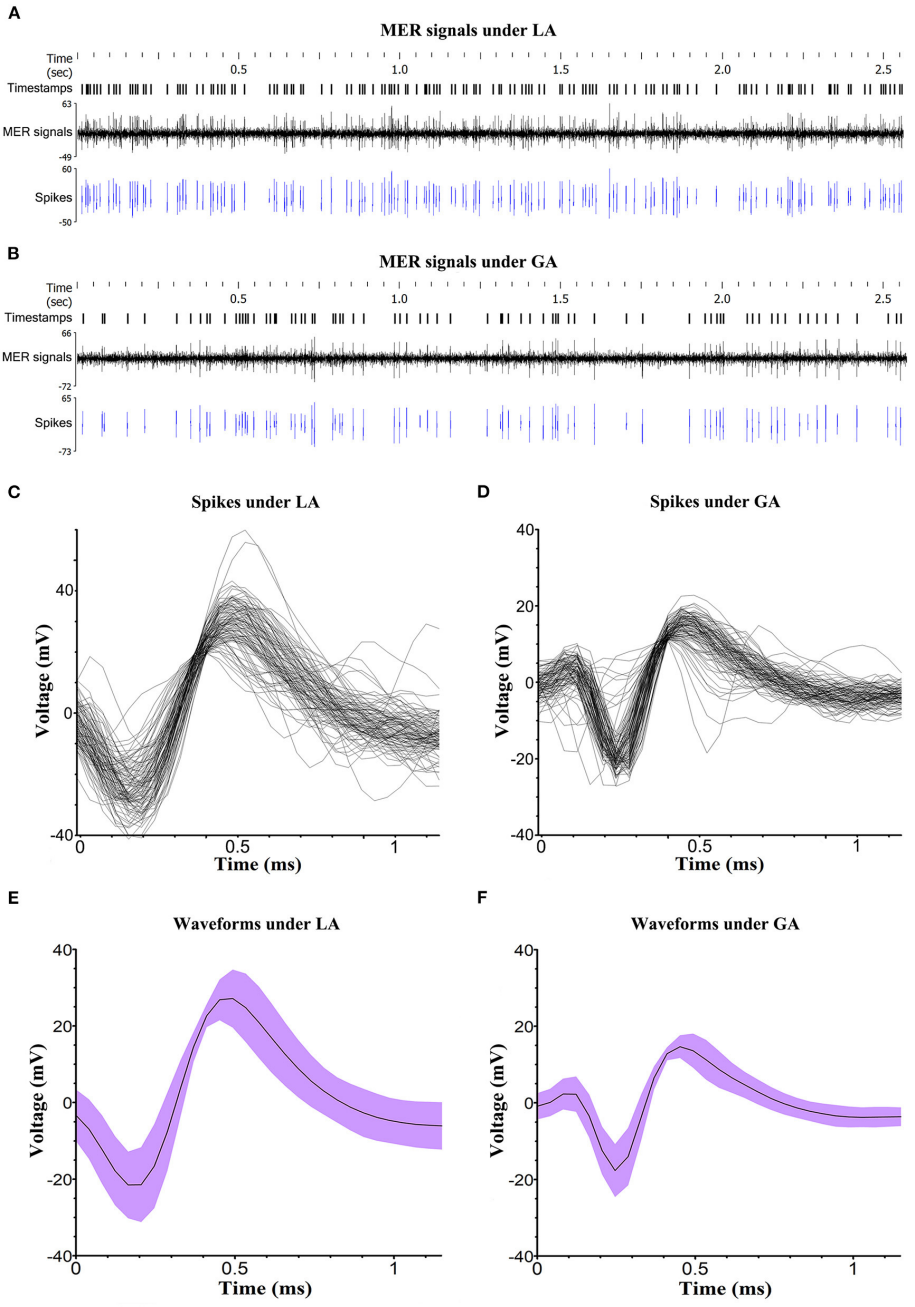
Analysis of MER signals. **(A, B)** Typical STN MER signals under LA and GA. The signal activities that were significantly over the baseline amplitude of background noise were selected and presented as spikes. Timestamps marked the specific time of detected spikes. **(C, D)** The spike sorting for a single unit in LA and GA groups, performed by NeuroExplorer (version 5.311). **(E, F)** The waveforms of spike under LA and GA. The voltage was displayed as mean value and deviation.

Altogether, there were 80 quadripolar DBS electrodes implanted in the STN of PD patients. The typical STN MER signals under LA and GA were analyzed and presented in Figure 2. In comparison with LA group, STN MER signals in GA group exhibited a decreased frequency (LA 45.4 ± 17.8 Hz vs. GA 34.4 ± 19.3 Hz, *P* < 0.05) and an increased ISI (LA 25.4 ± 11.1 ms vs. GA 39.8 ± 26.3 ms, *P* < 0.05). Meanwhile, the amplitudes of STN MER signals in GA group were reduced, with the minimum of waveform increased (LA −21.1 ± 9.5 mv vs. GA −14.3 ± 9.0 mv, *P* < 0.05) and the maximum of waveform decreased (LA 27.8 ± 11.5 mv vs. GA 11.6 ± 7.0 mv, *P* < 0.05). Whereas, the length of STN showed no statistically significant difference between LA and GA groups (LA 5.3 ± 0.7 mm vs. GA 5.2 ± 0.7 mm, *P* > 0.05) (Table 2).

**Table 2:** MER signal analysis of STN.

	**LA (*n* = 36)**	**GA (*n =* 44)**	***P*** **-value**
Frequency (Hz), mean±SD	45.4 ± 17.8	34.4 ± 19.3	0.01^*^
ISI (ms), mean±SD	25.4 ± 11.1	39.8 ± 26.3	0.00^*^
Min of Waveform (mv), mean±SD	−21.1 ± 9.5	−14.3 ± 9.0	0.00^*^
Max of Waveform (mv), mean±SD	27.8 ± 11.5	11.6 ± 7.0	0.00^*^
Length of STN (mm), mean±SD	5.3 ± 0.7	5.2 ± 0.7	0.88

MER, microelectrode recording; STN, subthalamic nucleus; ISI, inter-spike interval.

The symbol

* *p* < 0.05 and there are statistically significant differences.

### Electrode implantation accuracy

Vector errors (ΔX=|X_1_-X_2_|, ΔY=|Y_1_-Y_2_|, ΔZ=|Z_1_-Z_2_|, mm) and Euclidean distance ([ΔX^2^ + ΔY^2^ + ΔZ^2^]^1/2^, mm) were employed to assess the distance between the electrode implantation site to the intended target site. As shown in Table 3, vector errors were (0.53 ± 0.30 mm, 0.52 ± 0.39 mm, 0.40 ± 0.33 mm) and (0.54 ± 0.39 mm, 0.47 ± 0.36 mm, 0.54 ± 0.41 mm) in LA group and GA group respectively. The Euclidean distances were 0.95 ± 0.38 mm in LA group and 1.03 ± 0.43 mm in GA group. There was no statistically significant difference between LA and GA groups in Vector errors and Euclidean distance (*P* > 0.05). Therefore, similar electrode implantation accuracy was achieved in LA and GA groups.

**Table 3:** Electrode implantation accuracy.

	**LA (*n =* 36)**	**GA (*n =* 44)**	***P*** **-value**
**Vector errors (mm), mean**±**SD**			
ΔX	0.53 ± 0.30	0.54 ± 0.39	0.84
ΔY	0.52 ± 0.39	0.47 ± 0.36	0.60
ΔZ	0.40 ± 0.33	0.54 ± 0.41	0.10
Euclidean distance (mm), mean±SD	0.95 ± 0.38	1.03 ± 0.43	0.37

Vector error: ΔX=| X_1_-X_2_ |, ΔY=| Y_1_-Y_2_ |, ΔZ=| Z_1_-Z_2_ |; Euclidean distance = (ΔX^2^+ΔY^2^ +ΔZ^2^)^1/2^.

### Clinical outcome

Postoperative clinical assessment was performed in 6 months. UPDRS (-I, -II, -III, -IV) was applied to assess the motor and non-motor symptoms under on-DBS/off-medication condition. Actually, significant improvement of motor and non-motor symptoms with UPDRS (-I, -II, -III, -IV) scores declined were observed in both LA and GA groups. However, changes in UPDRS (-I, -II, -III, -IV) scores at 6 months from baseline were similar between LA and GA groups. Likewise, there were no statistically significant differences in the separate subscores of tremor, rigidity, bradykinesia, postural instability and gait disturbance (PIGD) between LA and GA groups. Postoperative LEDD was also significantly reduced at 6 months and the reduction of LEDD were similar between LA and GA groups (LA −805.6 ± 453.4 mg/day vs. GA −988.6 ± 507.1 mg/day, *P* > 0.05). Furthermore, patients in GA group achieved lower VAS scores (LA 5.6 ± 1.7 vs. GA 3.3 ± 1.1, *P* < 0.05) and higher GCQ scores (LA 54.7 ± 9.5 vs. GA 63.3 ± 8.9, *P* < 0.05) than those in LA group, indicating that patients experienced less pain and more comfort in GA group. Unexpectedly, the duration of surgery was similar between LA and GA groups (LA 222.6 ± 44.4 min vs. GA 211.0 ± 30.9 min, *P* > 0.05) (Table 4).

**Table 4:** Clinical outcome of MER guided STN-DBS surgery.

**Outcome**	**Baseline**		**Postoperative 6 months**		**Change at 6 months from baseline**		
	**LA**	**GA**	**LA**	**GA**	**LA**	**GA**	***P*** **-value**
UPDRS-I, mean±SD	12.2 ±3.0	10.9 ± 3.7	9.8 ±3.8	8.7 ± 3.3	−2.4 ± 2.0	−2.2 ± 1.8	0.73
UPDRS-II, mean±SD	18.1 ± 5.1	15.7 ± 5.5	11.7 ± 4.3	11.2 ± 4.8	−6.4 ± 3.7	−4.6 ± 2.5	0.07
UPDRS-III, mean±SD	50.2 ± 12.4	51.5 ± 11.3	33.2 ± 8.9	31.7 ± 8.6	−17.1 ± 7.0	−19.8 ± 10.2	0.34
Tremor, mean±SD	12.0 ± 2.7	12.6 ± 3.7	1.1 ± 0.9	1.6 ± 1.4	−10.9 ± 2.7	−10.9 ± 3.0	0.97
Rigidity, mean±SD	7.6 ± 2.5	7.3 ± 2.7	1.2 ± 0.7	0.9 ± 0.8	−6.4 ± 2.6	−6.5 ± 2.5	0.99
Bradykinesia, mean±SD	22.8 ± 5.1	21.7 ± 5.5	11.4 ± 3.2	10.4 ± 2.6	−11.4 ± 3.3	−11.3 ± 5.0	0.93
PIGD, mean±SD	2.9 ± 1.3	2.8 ± 1.2	1.2 ± 0.8	1.1 ± 0.9	−1.7 ± 1.0	−1.6 ± 1.0	0.79
UPDRS-IV, mean ± SD	10.1 ± 4.0	8.3 ± 3.6	3.9 ± 2.1	3.0 ± 2.0	−6.2 ± 3.0	−5.4 ± 2.7	0.38
LEDD (mg/day), mean±SD	1491.7 ± 393.4	1527.3 ± 413.1	686.1 ± 335.1	538.6 ± 264.5	−805.6 ± 453.4	−988.6 ± 507.1	0.24
VAS, mean±SD	5.6 ± 1.7	3.3 ± 1.1	NA	NA	NA	NA	0.00^*^
GCQ, mean±SD	54.7 ± 9.5	63.3 ± 8.9	NA	NA	NA	NA	0.01^*^
Duration of surgery, mean±SD	222.6 ± 44.4	211.0 ± 30.9	NA	NA	NA	NA	0.34

UPDRS, unified Parkinson's disease rating scale; PIGD, postural instability and gait disturbance; LEDD, levodopa equivalent daily dose; VAS, visual analog scale; GCQ, general comfort questionnaire.

The symbol

* *p* < 0.05 and there are statistically significant differences.

### Adverse events

There were no hardware-related postoperative adverse events occurred in this study. However, both LA and GA groups had one patient experienced non-symptomatic intracerebral hemorrhage. Two patients in GA group were diagnosed as postoperative pulmonary infection. No surgical site infection happened in both groups. In addition, several stimulation-related complications occurred in this study. For example, each group had one patient suffering from mild depression, one patient in LA group and two patients in GA group endured insomnia, and one patient in GA group complained of knee pain postoperatively.

## Discussion

Since DBS surgery was introduced by Benabid and his colleagues in 1987, this technique had experienced continuous improvement (13). Currently, MER guided STN-DBS under LA has become the preferred therapeutic method for advanced PD patients with motor fluctuation and dyskinesia after long-term medication (3, 13). Since awake DBS under LA is painful and burdensome for PD patients, more and more neurosurgeons have tried to perform MER guided STN-DBS under GA (1). GA helps to alleviate anxiety, ease pain and improve cooperation during the entire surgical procedures (14, 15). However, the debate is still ongoing concerning the influence of GA on MER signals, electrode implantation accuracy, clinical outcome and adverse events in MER guided STN-DBS.

### MER signals

The effect of general anesthesia on MER is controversial but likely depends on the type and dose of a particular anesthetic agent, underlying disease, and surgical target (14, 16–18). The commonly used anesthetic agents in DBS are propofol, remifentanil, fentanyl, desflurane, sevoflurane, isoflurane and dexmedetomidine (17). Among them, propofol is a short-acting, easily titratable and very predictable drug that has been reported in many centers for general anesthesia or sedation during DBS surgery (18). Propofol functions by activating gamma-aminobutyric acid (GABA) receptors, thus its influence on MER may depend on the number of GABAergic neurons in the selected target. The lateral and medial globus pallidus and substantia nigra are mainly constituted by GABAergic neurons, while STN is mainly constituted by glutamatergic neurons (19–21). Therefore, propofol may have little effect on MER in STN-DBS. However, whether intraoperative MER guided STN DBS under general anesthesia with propofol is feasible and effective? It remains controversial. Hertel et al. showed that propofol changed the typical background activity of the STN (16). Maltete et al. found that the electrode implantation accuracy and clinical outcomes of DBS under general anesthesia with propofol were inferior to those achieved without anesthesia (22). Whereas, Maciver et al. (23) demonstrated that both propofol and remifentanil produced only minor alterations of subthalamic neuron discharge activity that should not interfere with DBS surgery (23).

In this study, propofol and remifentanil were applied to implement GA and BIS value was maintained at 40–60 during MER. Under the well-defined circumstance, the bursting firing pattern of STN was typical (14, 16). Although, the parameters of STN MER signal, including frequency, ISI and amplitude, were obviously interfered under GA, the waveforms of STN MER signal were recognizable and shared similar characteristics in LA and GA groups. Therefore, intraoperative MER was a viable method to localize STN under GA.

### Electrode implantation accuracy

In addition to the properly selected candidates, precise implantation of the electrodes in STN is the most important factor in achieving expected clinical outcomes (14). However, multiple factors might have influences on electrode implantation accuracy, including errors in image fusion, manipulation errors from instrument, brain shift due to CSF leakage and electrode displacement during MRI scanning (24–26). In this study, we employed Vector errors and Euclidean distance to evaluate the electrode implantation accuracy. Intriguingly, the results showed no statistically significant differences in Vector errors and Euclidean distance between LA and GA groups. Furthermore, the electrode implantation accuracy in this study was comparable to the results of other studies, even to the results of MRI-guided STN-DBS studies (27, 28). The length of STN is another important parameter indicating the suitable target. Generally, the trajectory that intersects with STN over 4 mm, is recommended as the ideal microelectrode trajectory (29). We also got similar length of STN in LA and GA groups. Moreover, the length of STN in both groups exceeded 4 mm. These results indicated that electrodes were implanted into suitable target sites in both groups. Consequently, LA and GA can achieve similarly effective electrode implantation accuracy in MER guided STN-DBS surgery.

### Clinical outcome

In both LA and GA groups, PD patients were benefit from STN-DBS. According to the assessment of UPDRS (-I, -II, -III, -IV), the improvement of motor and non-motor symptoms after bilateral STN-DBS under GA was comparable with the improvement achieved under LA. Likewise, the separate subscores of tremor, rigidity, bradykinesia and PIGD achieved similar reduction in LA and GA groups. These findings matched the results of previous studies, which compared the clinical outcome of STN-DBS under different anesthetic methods (30–32). The reduction of LEDD at 6 months postoperatively showed no statistically significant differences between LA and GA groups. Rozemarije A. Holewijn also drew a similar conclusion in a randomized clinical trial (3). In addition, GA enabled patients to have a better surgical experience with less pain and more comfort. In comparison with LA, several procedures of MER guided STN-DBS surgery were simplified under GA, including test stimulation (3, 16). However, in this study, there was no statistically significant difference in the duration of surgery between LA and GA groups. Owning to the individual difference in anesthetic metabolism, it usually took extra time to maintain BIS value at 40–60 during MER in GA group. With the experience accumulating, the duration of surgery in GA group is expected to be shorten. Thus, both LA and GA can achieve expected clinical outcome in MER guided STN-DBS surgery.

### Adverse events

**Figure 3:**
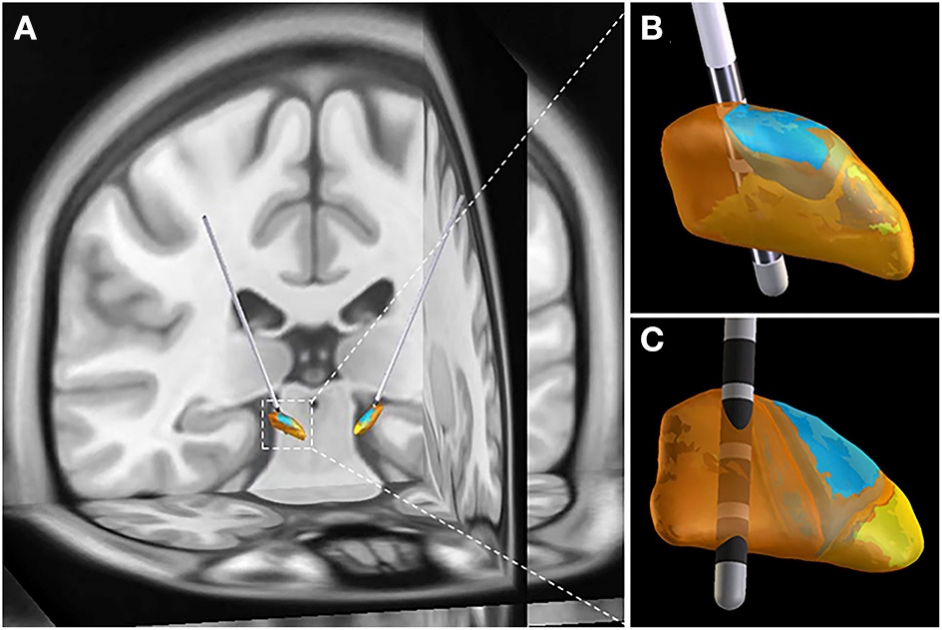
Representative case with electrodes implanted into sensorimotor STN. **(A)** 3-D image reconstructed by preoperative MRI image and postoperative CT image. **(B)** Anterior view of STN. **(C)** Posterior view of STN. STN is partitioned into sensorimotor (orange), limbic (yellow) and associative (blue) subregions. The image reconstruction was performed by Lead-DBS.

The postoperative adverse events were categorized as hardware-related, surgery-related, and stimulation-related complications. No hardware-related postoperative adverse events occurred in this study. However, the incidence of intracerebral hemorrhage was 5%, which was consistent with the overall risk of intracerebral hemorrhage reported before (33, 34). It seemed that more patients in GA group experienced postoperative pulmonary infections, which might be owing to GA and intubation during the surgical procedures. Actually, most postoperative adverse events were stimulation-related complications in this study. STN is usually partitioned into sensorimotor, limbic and associative subregions (Figure 3). The stimulation-related adverse events can be reduced by targeting sensorimotor STN (35–40). Thus, the electrode implantation accuracy directly influences the surgical outcomes. Advances in radiological and neural electrophysiological techniques, which contribute to improving electrode implantation accuracy, are expected to propel the development of this discipline.

### Limitations

There are several limitations in this present study. Firstly, the surgery was performed under the guideline of frame-based MER. The applications of these results are limited in MRI guided STN-DBS or frameless STN-DBS surgery. Secondly, the BIS value recommended for GA remains controversial. To determine the optimal BIS value for GA in MER guided STN-DBS, more studies are necessary. Finally, the patients enrolled in this study was limited and the analysis was performed retrospectively.

## Conclusion

GA is viable and effective in MER guided STN-DBS. Although, the parameters of STN MER signals, including frequency, ISI and amplitude, were obviously interfered under GA, the waveforms of MER signals were recognizable and shared similar characteristics with LA group. GA was also comparable with LA in MER guided STN-DBS, regarding electrode implantation accuracy, clinical outcome and adverse events. Notably, GA is more friendly and acceptable to the patients who are incapable of enduring intraoperative MER under LA.

## Data availability statement

The original contributions presented in the study are included in the article/Supplementary material, further inquiries can be directed to the corresponding author.

## Ethics statement

Written informed consent for surgery was obtained from all patients and the anesthesia methods were determined on the basis of patients' preferences. The demographics and baseline information of patients were acquired from medical records with their approval.

## Author contributions

PF and KQ conceived of the paper. KQ wrote the initial draft of the paper and generated the figures and tables. JW and JR collected the medical records and data. PZ, YS, WH, and JH performed the MER signal analysis. XJ edited and revised the manuscript. All authors contributed to the article and approved the submitted version.
