# Supplementary material for: Intraoperative microelectrode recording under general anesthesia guided subthalamic nucleus deep brain stimulation for Parkinson's disease: One institution's experience
Source: Front Neurol. 2023 Feb 23;14:1117681. doi: 10.3389/fneur.2023.1117681 (PMC9997081; doi:10.3389/fneur.2023.1117681)
Supplement: Supplementary file 1 [file Table_1.docx]

**Supplementary table 1.** Characteristics of the patients and reasons for general anesthesia.

| Patient No. | Gender | Age (year) | Duration of PD (year) | UPDRS-III (med off) | H&Y stage (med off) | MMSE (med off) | LEDD (mg/day) | Anesthesia | Reason for GA |
| --- | --- | --- | --- | --- | --- | --- | --- | --- | --- |
| 1 | F | 63 | 5 | 66 | 4 | 28 | 1800 | LA | NA |
| 2 | F | 59 | 4 | 61 | 3 | 27 | 1500 | LA | NA |
| 3 | F | 67 | 10 | 46 | 2 | 28 | 1500 | GA | Anxiety, fear |
| 4 | F | 58 | 7 | 55 | 3 | 28 | 900 | LA | NA |
| 5 | F | 66 | 6 | 38 | 2 | 25 | 900 | GA | Insufficient cooperation, fear |
| 6 | F | 65 | 4 | 60 | 3 | 29 | 1500 | GA | Vertebral pain |
| 7 | F | 66 | 8 | 35 | 3 | 27 | 1800 | LA | NA |
| 8 | M | 60 | 9 | 47 | 2 | 29 | 2400 | LA | NA |
| 9 | F | 65 | 6 | 63 | 3 | 26 | 1800 | GA | Severe convulsions |
| 10 | F | 65 | 7 | 35 | 2 | 25 | 1200 | GA | Language difficulties |
| 11 | F | 63 | 8 | 63 | 2 | 28 | 1800 | GA | Vertebral pain, fear |
| 12 | M | 61 | 10 | 32 | 2 | 30 | 1800 | LA | NA |
| 13 | F | 63 | 13 | 40 | 2 | 29 | 1500 | GA | Anxiety, fear |
| 14 | M | 50 | 7 | 68 | 3 | 29 | 1500 | LA | NA |
| 15 | F | 60 | 6 | 39 | 3 | 28 | 1200 | LA | NA |
| 16 | F | 58 | 5 | 39 | 3 | 30 | 1500 | GA | Fear, severe convulsions |
| 17 | M | 71 | 8 | 45 | 3 | 29 | 1500 | GA | Insufficient cooperation, fear |
| 18 | F | 69 | 9 | 59 | 4 | 30 | 1200 | LA | NA |
| 19 | F | 63 | 7 | 43 | 3 | 28 | 1800 | GA | Anxiety, fear |
| 20 | M | 72 | 12 | 56 | 3 | 30 | 2400 | GA | Difficult breathing, fear |
| 21 | M | 71 | 8 | 65 | 3 | 28 | 1800 | LA | NA |
| 22 | F | 75 | 13 | 46 | 2 | 28 | 1500 | LA | NA |
| 23 | M | 58 | 5 | 71 | 4 | 27 | 1200 | GA | Insufficient cooperation |
| 24 | M | 58 | 5 | 43 | 2 | 28 | 1800 | GA | Anxiety, fear |
| 25 | M | 46 | 9 | 61 | 3 | 29 | 1500 | GA | Vertebral pain |
| 26 | F | 65 | 8 | 37 | 2 | 30 | 1200 | LA | NA |
| 27 | M | 42 | 8 | 46 | 2 | 28 | 900 | GA | Insufficient cooperation, fear |
| 28 | F | 67 | 11 | 57 | 2 | 30 | 2400 | GA | Severe convulsions |
| 29 | F | 55 | 13 | 58 | 2 | 29 | 1200 | GA | Anxiety, fear |
| 30 | M | 70 | 13 | 31 | 3 | 29 | 900 | LA | NA |
| 31 | F | 57 | 10 | 71 | 3 | 28 | 1500 | GA | Vertebral pain, fear |
| 32 | F | 58 | 5 | 59 | 4 | 28 | 1800 | GA | Fear, coughing |
| 33 | F | 67 | 8 | 63 | 3 | 28 | 1050 | LA | NA |
| 34 | F | 64 | 10 | 38 | 2 | 26 | 900 | GA | Anxiety, fear |
| 35 | M | 63 | 7 | 55 | 2 | 29 | 1800 | LA | NA |
| 36 | F | 63 | 10 | 59 | 3 | 28 | 1200 | GA | Vertebral pain |
| 37 | F | 52 | 5 | 41 | 2 | 29 | 1800 | GA | Anxiety, fear |
| 38 | M | 64 | 6 | 56 | 3 | 29 | 1500 | LA | NA |
| 39 | M | 61 | 9 | 39 | 2 | 28 | 1200 | LA | NA |
| 40 | F | 56 | 7 | 50 | 3 | 29 | 1800 | LA | NA |

*UPDRS-III, unified Parkinson’s disease rating scale III; H&Y stage, Hoehn and Yahr stage; MMSE, mini mental state examination; LEDD, levodopa equivalent daily dose. F, female; M, male; LA, local anesthesia; GA, general anesthesia.*
